# Associated factors with dietary patterns among children under 2 years of age: a study in childcare centres and homes of South Brazil

**DOI:** 10.1017/jns.2021.26

**Published:** 2021-05-14

**Authors:** Juliana R. D. Guedes, Doroteia A. Höfelmann, Fernanda P. Madruga, Elaine C. V. de Oliveira, Mônica M. O. de Cerqueira, Alline C. S. Lobo, Cláudia C. B. Almeida

**Affiliations:** 1 Department of Nutrition, Federal University of Paraná (UFPR), Curitiba 80210-170, Brazil; 2 Department of Nutrition, Federal University of Pernambuco (UFPE), Recife 50670-901, Brazil

**Keywords:** Children, Complementary feeding, Dietary patterns, Food consumption, Infant feeding

## Abstract

Identifying dietary patterns in different environments attended by children is relevant to guide public politics. The aim of this study was to analyse the factors associated with dietary patterns of children under 2 years of age in childcare centres and at homes. This transversal study was enrolled in municipal childcare centres of Guaratuba, Paraná, Brazil. Food consumption data from 256 children were obtained by the food record method. From the consumption data, four dietary patterns were identified by factor analysis using the principal component method in each environment. ‘Traditional’ patterns were observed in both environments; the ‘less healthy’ pattern was found only at homes. Other patterns identified in childcare centres include ‘snacks’, ‘nutritive’ and ‘pasta and meats’; at homes, it was possible to identify patterns such as ‘milk and cereals’ and ‘mixed’. Children over 12 months presented higher scores for all the patterns in both environments. Obese children had lower scores for all the patterns in childcare centres. There was an association between maternal age below 21 years and higher adherence to a ‘less healthy’ pattern, maternal level of education less than 8 years and lower adherence to the ‘mixed’ pattern and lower familiar income per capita and higher adherence to the ‘snacks’ and ‘traditional’ patterns in childcare centres. In conclusion, adherence to dietary patterns was associated with socio-economic, demographic variables and nutritional status and further studies are needed, especially those with a longitudinal design, enabling the monitoring of dietary patterns.

Epidemiologist studies have become common to make the use of dietary patterns to evaluate food consumption, since such studies consider the habitual feeding in a global form and recognise that people consume a combination of both food and nutrients^(1)^. Studies also indicate that dietary patterns in the first years of life are maintained during infancy^(2–4)^ and can remain steady until adult life^(5)^.

Scientific evidence has demonstrated the association between dietary patterns of children and their impacts on theirs health, such as the presence of respiratory disease symptoms^(6)^, effects in the intelligence quotient^(7,8)^, cognitive ability^(9)^, modification of bone mineral density^(10)^, alteration of corporal composition^(2,11,12)^ and obesity^(13)^.

Childhoods’ feeding is directly affected by the social environment^(14–16)^. At homes, children tend to follow less healthy dietary patterns, especially when they are inserted in a family context filled with vulnerabilities, including low income and lower levels of education of the parents^(15)^.

Educational institutions, in which children receive health and feeding cares, also play an important role in food consumption^(16)^. Scholar feeding programmes aim to improve the school performance and learning by supplying meals^(17)^. Thus, the school environment can assist the promotion of healthier eating habits^(18)^. However, some studies recognised the need to adequate the scholar meals, so that they can reach the nutritional requirements recommended by the specific legislation^(19,20)^.

There are few studies that have simultaneously investigated the food consumption of children in those two environments, school and home^(21)^, and only one has evaluated dietary patterns in both cases^(22)^. It is relevant to identify dietary patterns in different environments attended by the children in order to guide public politics and to allow the development of educational actions on health and nutrition^(22)^. The objectives of this study were to identify the dietary patterns of children under 2 years of age in childcare centres and at homes and to analyse the association between socio-economic and demographic conditions and the nutritional status.

## Methodology

### Delineation and population of the study

This is a cross-sectional and analytical study carried out in Guaratuba, Paraná, Brazil, from February to September 2014, which is part of the ‘Alimentary and Nutritional Security in School Environment’ project. Further information on this project can be found elsewhere^(23,24)^.

All children of both sexes under 2 years of age were considered eligible to participate in the study. They had to be enrolled and had to attend all the five municipal childcare centres of Guaratuba during the data collection period, except exclusively breastfed children or the ones following special diets. The parents or caregivers had to sign the Written Informed Consent Form. This study was conducted according to the guidelines laid down in the Declaration of Helsinki, and all procedures involving human subjects were approved by the Committee of Ethics in Research of the Sector of Sciences of the Health of the Federal University of the Paraná (11312612.5.0000.0102).

There were nineteen losses (6⋅9 %) out of 275 eligible children: one for not having the weighted food record data; ten for not bringing back the estimated food record; one for not fulfilling correctly the estimated food record and seven for not having the authorisation of the parents or caregivers to participate in the study, which resulted in a total of 256 children.

### Anthropometrics measures

Weight and length measures were taken in duplicates by trained researchers. To achieve these, children wore the minimum of clothes possible and were bare-footed. Weight was measured in grams by a pediatric Plenna® scale of Tinn 00038 model with a capacity of 15 kg and 10 g graduation; length was measured in centimetres through a Tonelli® stadiometer with an amplitude of measure from 0 to 1000 mm.

The nutritional status classification was performed using WHO Anthro® software version 3.2.2. The *Z*-score of body mass index was based on age, body mass index by age (BMI/A), and divided into four categories: underweight (<−2); normal weight (≥−2 and ≤+1); overweight (>+1 and ≤+2) and obesity (>+2)^(25)^.

### Socio-economic and demographic variables

A structuralised and previously tested questionnaire of socio-economic and demographic data was administered to the parents and the caregivers of the children. The following variables were considered: sex (male/female); age in months (<12; ≥12); birth weight in grams (<2500; ≥2500 and <4000; ≥4000); breast-feeding (yes/not); age of the mother in years (≤20; >20 and ≤30; >30); level of education of the parents in years (<8; ≥8 and <11; ≥11) and familiar income per capita in the minimum wage, effective in the period of data collection, R$724⋅00/US$321⋅80^(26)^, divided in tertiles (≤0⋅35; >0⋅35 and <0⋅70; ≥0⋅70).

### Food consumption evaluation

Data related to food consumption were obtained by means of the food record method (estimated and weighted)^(27)^. The weighted food record was collected by the field team for all the meals offered during the permanence of the children in childcare centres in two non-consecutive days (weekdays). To quantify the total food consumed by the children, the repetitions were added to the amount served, and the remnant portions were subtracted from the meals. A portable digital Plenna® scale with a capacity of 5 kg and 1 g precision was used for food weighing. A measuring cylinder with a capacity of 250 and 10 ml graduation was used in order to quantify the liquids.

A 3-d estimated food record was filled by the parents and caregivers in order to collect information about the meals that the children had at homes, from the moment which they left the childcare centres until the moment which they returned on the following day, as well as one weekend day, specifically on Sundays. The estimated food record provided illustrations of utensils, referred to the type and the size, to assist the parents or caregivers to estimate the size of the portions during the meals. Intending to achieve the amount of breast milk consumed, an average breast milk consumption guide for children in complementary feeding was considered according to the age and the recommendation of the World Health Organization^(28)^.

Out of 256 children, 252 (98⋅4 %) presented data related to 2 d in the childcare centres of weighted food record and at homes from estimated food record; 206 (80⋅5 %) had proceeding data from 3 d, and twenty-five (9⋅8 %) of 2 d. It was possible to obtain seventy-eight different foods from the childcare centres and 191 foods from homes. After that, the foods were divided based on nutritional similarity degrees, culinary use and consumption frequency. The groups were named according to the food items in them. A total of twenty-one and twenty-three groups related to childcare centres and the homes were created, respectively. The food groups’ composition presented small differences between the environments, once foods taken at home showed a greater variety in relation to the ones taken in the childcare centres.

Adjustments in intra- and inter-individual variability related to food quantification in the groups, which were categorised by the environments, were performed in order to obtain the usual consumption by the multiple source method. This method esteems the usual consumption by means of a procedure of three steps. First, the probability of an individual consuming a given food or nutrient on a random day is estimated. Secondly, the usual amount of food or nutrient ingestion in 1 d of consumption is esteemed. Thirdly, the final numbers resulting from those two steps are multiplied by each other to estimate the usual daily ingestion for each individual. From the individual calculation, the method constructs the food consumption distribution of the studied population^(29)^.

Food groups, which presented low correlation with other groups (Spearman's correlations less than 0⋅20), and, therefore, not reached the patterns, were excluded from the analyses. Accordingly, three food groups were excluded: one proceeding from childcare centres (‘breast milk’) and two from homes (‘soups’ and ‘natural juice’), thus resulting in twenty and twenty-one groups, respectively.

### Statistical analyses

Dietary patterns were derived by means of factor analysis using the principal component method distinct for each environment. The tests by the Sphericity of Bartlett and Kaiser–Mayer–Olkin coefficient^(30)^ were used to evaluate the adequacy of the sample, being considered acceptable a value of *P* ≤ 0⋅05 and a coefficient of >0⋅6, respectively. This study also considered the eigenvalue criterion >1, the scree plot^(31)^ test, and the interpretability of the components in order to determine the number of factors to be extracted. Food groups with factor loadings of >0⋅30 or <−0⋅30 were considered in order to compose each dietary pattern. The factors were submitted to Varimax^(32)^ orthogonal transformation for greater data interpretability. The internal consistency between the items of each pattern was evaluated through Cronbach's α coefficient^(33)^. The dietary patterns identified were named in accordance with the characteristics of the food groups with higher factor loadings.

Scores for each factor were generated in order to verify the adherence of the children to the dietary patterns. Such scores were calculated by the addition of the standardised values in the Z-score of the food groups of each pattern and were weighted according to their respective factor loading. A higher score indicates a higher adherence to the respective dietary pattern. Values for each factor were categorised into tertiles to represent three levels of adherence to each specific dietary pattern. Associations between the tertiles and the socio-economic and demographic conditions, as well as nutritional status, were investigated by ordered logistic regression, expressed by proportional odds ratio (OR) of being at the higher tertile of adherence, compared with the bottom, and its 95 % confidence intervals (95 % CI).

Statistical analyses were performed by Stata® software, version 14. A significance level of 5 % was considered.

## Results

Table 1 shows the population characterisation of the study. More than half of the children were male (57⋅0 %), and the majority had between 12 and 24 months of age (71⋅1 %).

**Table 1.** Characterisation of the population of the study, children under 2 years of age who attended municipal childcare centres in Guaratuba, Paraná, Brazil, 2014 (number of participants and percentage, *n* 256)

Variable	*N*	%
Sex		
Male	146	57⋅0
Female	110	43⋅0
Age (months)		
≥4 and <12	74	28⋅9
≥12 and <24	182	71⋅1
Birth weight (g)		
<2500	20	7⋅8
≥2500 and <4000	205	80⋅1
≥4000	25	9⋅8
NI	6	2⋅3
Current breast-feeding situation		
Yes	88	34⋅4
Not	162	63⋅3
NI	6	2⋅3
Age of the mother (years)		
≤20	51	19⋅9
>20 and ≤30	118	46⋅1
>30	83	32⋅4
NI	4	1⋅6
Education level of the mother (years)		
<8	57	22⋅2
≥8 and <11	77	30⋅1
≥11	118	46⋅1
NI	4	1⋅6
Family income per capita^a^		
≤0⋅35	74	28⋅8
>0⋅35 and <0⋅70	77	30⋅1
≥0⋅70	68	26⋅6
NI	37	14⋅5
Nutritional status^b^		
Underweight	4	1⋅6
Normal weight	148	57⋅8
Overweight	53	20⋅7
Obesity	46	18⋅0
NI	5	1⋅9
Childcare centres		
01	30	11⋅7
02	54	21⋅1
03	25	9⋅8
04	81	31⋅6
05	66	25⋅8

NI, not informed.

a Family income per capita divided in tertiles – according to minimum wage effective in 2014 – of R$724⋅00/US$321⋅80^(26)^.

b Nutritional status regarding body mass index by age (BMI/A).

Four dietary patterns were observed in each environment, which could explain 58⋅8 and 40⋅2 % of the total variance in the childcare centres and at homes, respectively. These patterns are presented in Tables 2 and 3.

**Table 2.** Dietary patterns in childcare centres according to the factorial analysis using the principal component method for children under 2 years of age in municipal childcare centres in Guaratuba, Paraná, Brazil, 2014 (factorial loads >0⋅3 and <−0⋅3)

Dietary patterns	Food items	Factor loading	% explained variance	Cronbach's α
Pattern 1 ‘Snacks’	Margarine and butter	0⋅78	27⋅5	0⋅74
	Coffee and teas	0⋅74		
	Biscuits and breads	0⋅66		
	Fruits	0⋅63		
	Formula	−0⋅57		
Pattern 2 ‘Nutritive’	Natural juice	0⋅88	13⋅9	0⋅77
	Tubercles, roots and cereals	0⋅73		
	Vegetables	0⋅67		
	Dairy products	0⋅53		
	Breakfast cereals	−0⋅55		
Pattern 3 ‘Pasta and meats’	Sauce	0⋅76	10⋅0	0⋅72
	Meats and eggs	0⋅74		
	Dainties	0⋅72		
	Pasta	0⋅54		
Pattern 4 ‘Traditional’	Milk	0⋅74	7⋅4	0⋅69
	Processed meats	0⋅66		
	Beans	0⋅59		
	Rice	0⋅57		
	Sugar	0⋅49		
	Soup	−0⋅53		
Total			58⋅8

**Table 3.** The dietary patterns observed at home according to the factorial analysis using the principal component method for children under 2 years of age in municipal childcare centres in Guaratuba, Paraná, Brazil, 2014 (factorial loads >0⋅3 and <−0⋅3)

Dietary patterns	Food items	Factor loading	% explained variance	Cronbach's α
Pattern 1 ‘Traditional’	Rice	0⋅76	16⋅8	0⋅62
	Meats, eggs and fish	0⋅71		
	Beans	0⋅60		
	Fruits	0⋅38		
	Sauce	0⋅37		
	Pasta	0⋅35		
Pattern 2 ‘Less healthful’	Processed meats	0⋅60	8⋅7	0⋅64
	Dainties	0⋅57		
	Sugar drinks	0⋅57		
	Sugar	0⋅57		
	Biscuits, breads and cakes	0⋅46		
	Pasta and salty	0⋅45		
	Margarine and similar	0⋅41		
	Dairy products	0⋅33		
Pattern 3 ‘Milk and cereals’	Milk	0⋅81	7⋅8	0⋅60
	Breakfast cereals	0⋅78		
	Breast milk	−0⋅65		
Pattern 4 ‘Mixed’	Coffee and teas	0⋅62	6⋅9	0⋅43
	Vegetables	0⋅59		
	Tubercles, roots and cereals	0⋅59		
	Formula	0⋅48		
Total			40⋅2	

Regarding childcare centres, the first pattern presented positive loadings for biscuits and breads, margarine and butter, coffee and teas, and fruits, with negative loading for formula, which was labelled the ‘snacks’ pattern. The second pattern is composed of natural juice, tubercles, roots and cereals, vegetables and dairy products. It presented negative loading for breakfast cereal and configured the ‘nutritive’ pattern. Furthermore, the ‘pasta and meats’ pattern is the third one, and it had positive loadings for pasta, sauce, meats and eggs, and dainties. The fourth pattern includes rice, beans, processed meats, milk and sugar; it had negative loading for soup, and it was called ‘traditional’ pattern (Table 2).

At homes, the first pattern presented positive loadings for rice, beans, meats, eggs and fish, fruits, pasta and sauce, being named as ‘traditional’ pattern. The second pattern had positive loadings for processed meats, dainties, sugar drinks, sugar, pasta and salty, biscuits, breads and cakes, margarine and similar, and dairy products. It was labelled ‘less healthy’ pattern. The third pattern was constituted by milk and breakfast cereal, and it also presented negative loadings for breast milk and received the name of ‘milk and cereals’ pattern. The fourth pattern was composed of coffee and teas, vegetables, tubercles, roots and cereals, and formula. This pattern was called ‘mixed’ pattern and was the only one whose internal consistency presented a Cronbach's α value below 0⋅6 (Table 3).

The four dietary patterns, in the childcare centres, were associated with the age of the child and nutritional status. In addition, the ‘snacks’ pattern showed an association with per capita family income and childcare centres in which the child was enrolled. The ‘nutritive’ pattern was associated with the birth weight and childcare centres, and the ‘traditional’ pattern showed an association with the current breast-feeding situation, per capita family income and childcare centres. Regarding the age, children older than 12 months presented higher odds of being at the highest quantile of adherence for all dietary patterns identified in the childcare centres. Furthermore, the odds of a higher level of adherence to the dietary patterns increase with nutritional status classification until overweight. On the other hand, obese children presented lower scores for all patterns in this environment. Breastfed children showed lower odds for ‘traditional’ patterns. Concerning childcare centres, number two had higher scores for the ‘nutritive’ pattern, number four had lower scores for ‘snacks’ and ‘nutritive’ patterns and number five showed lower scores for ‘snacks’, ‘nutritive’ and ‘traditional’ patterns. The children with higher birth weight had more odds of adherence to ‘nutritive’ pattern, and also those whose families had lesser per capita income had higher scores for the ‘snacks’ pattern (Table 4).

**Table 4.** Distribution of factor scores related to the dietary patterns found in the childcare centres according to socio-economic, demographic variables and nutritional status for children under 2 years of age in municipal childcare centres in Guaratuba, Paraná, Brazil, 2014 (Odds ratio and confidence interval; statistical significance *P* < 0⋅05)

Variables	Pattern 1^a^ ‘Snacks’ Odds ratio (95 % confidence intervals)	Pattern 2^a^ ‘Nutritive’ Odds ratio (95 % confidence intervals)	Pattern 3^a^ ‘Pasta and meats’ Odds ratio (95 % confidence intervals)	Pattern 4^a^ ‘Traditional’ Odds ratio (95 % confidence intervals)
Sex	*P* 0⋅825	*P* 0⋅393	*P* 0⋅947	*P* 0⋅826
Male (*n* 146)	1⋅00	1⋅00	1⋅00	1⋅00
Female (*n* 110)	1⋅05 (0⋅67–1⋅66)	0⋅82 (0⋅52–1⋅29)	1⋅02 (0⋅64–1⋅60)	1⋅05 (0⋅67–1⋅66)
Age of the child (months)	***P 0⋅000	*P 0⋅021	***P 0⋅000	***P 0⋅000
<12 (*n* 74)	1⋅00	1⋅00	1⋅00	1⋅00
≥12 (*n* 182)	8⋅34 (4⋅71–14⋅75)	1⋅81 (1⋅09–2⋅99)	2⋅66 (1⋅56–4⋅53)	7⋅66 (4⋅34–13⋅52)
Birth weight (g)	*P* 0⋅857	*P 0⋅029	*P* 0⋅997	*P* 0⋅584
<2500 (*n* 20)	1⋅00	1⋅00	1⋅00	1⋅00
≥2500 and < 4000 (*n* 205)	0⋅97 (0⋅43–2⋅15)	1⋅65 (0⋅71–3⋅86)	0⋅99 (0⋅42–2⋅33)	0⋅55 (0⋅24–1⋅26)
≥4000 (*n* 25)	1⋅08 (0⋅39–2⋅97)	3⋅32 (1⋅11–9⋅93)	1⋅00 (0⋅33–3⋅00)	1⋅15 (0⋅40–3⋅30)
Current breast-feeding situation	*P* 0⋅412	*P* 0⋅097	*P* 0⋅508	*P 0⋅048
Yes (*n* 88)	1⋅00	1⋅00	1⋅00	1⋅00
No (*n* 162)	1⋅22 (0⋅76–1⋅98)	1⋅50 (0⋅93–2⋅42)	1⋅18 (0⋅73–1⋅91)	1⋅62 (1⋅00–2⋅62)
Age of the mother (years)	*P* 0⋅831	*P* 0⋅736	*P* 0⋅406	*P* 0⋅617
≤20 (*n* 51)	1⋅00	1⋅00	1⋅00	1⋅00
>20 and ≤ 30 (*n* 118)	1⋅04 (0⋅57–1⋅91)	1⋅29 (0⋅70–2⋅38)	1⋅70 (0⋅91–3⋅19)	1⋅56 (0⋅86–2⋅82)
>30 (*n* 83)	1⋅07 (0⋅57–2⋅03)	1⋅17 (0⋅61–2⋅23)	0⋅90 (0⋅47–1⋅76)	0⋅93 (0⋅49–1⋅75)
Education level of the mother (years)	*P* 0⋅172	*P* 0⋅257	*P* 0⋅909	*P* 0⋅814
<8 (*n* 57)	1⋅00	1⋅00	1⋅00	1⋅00
≥8 and < 11 (*n* 77)	0⋅51 (0⋅27–0⋅96)	0⋅93 (0⋅49–1⋅75)	0⋅88 (0⋅46–1⋅67)	0⋅63 (0⋅34–1⋅16)
≥11 (*n* 118)	0⋅61 (0⋅34–1⋅09)	0⋅73 (0⋅41–1⋅31)	0⋅94 (0⋅52–1⋅70)	0⋅86 (0⋅49–1⋅54)
Family income per capita^b^	**P 0⋅009	*P* 0⋅322	*P* 0⋅682	**P 0⋅007
≤0⋅35 (*n* 74)	1⋅00	1⋅00	1⋅00	1⋅00
>0⋅35 and <0⋅70 (*n* 77)	0⋅67 (0⋅37–1⋅21)	0⋅79 (0⋅44–1⋅41)	1⋅03 (0⋅57–1⋅85)	0⋅60 (0⋅33–1⋅07)
≥0⋅70 (*n* 68)	0⋅44 (0⋅23–0⋅81)	0⋅74 (0⋅40–1⋅36)	0⋅88 (0⋅48–1⋅61)	0⋅43 (0⋅23–0⋅79)
Nutritional status^c^	***P 0⋅000	**P 0⋅009	*P 0⋅019	***P 0⋅000
Underweight (*n* 4)	1⋅00	1⋅00	1⋅00	1⋅00
Normal weight (*n* 148)	2⋅29 (0⋅33–15⋅65)	7⋅79 (0⋅82–73⋅79)	2⋅14 (0⋅32–14⋅47)	2⋅26 (0⋅33–15⋅62)
Overweight (*n* 53)	3⋅53 (0⋅49–25⋅31)	17⋅57 (1⋅77–174⋅11)	2⋅44 (0⋅35–17⋅11)	3⋅93 (0⋅55–28⋅32)
Obesity (*n* 46)	0⋅27 (0⋅04–2⋅09)	1⋅48 (0⋅15–15⋅01)	0⋅77 (0⋅11–5⋅52)	0⋅29 (0⋅04–2⋅18)
Childcare centres	***P 0⋅000	***P 0⋅000	*P* 0⋅097	**P 0⋅002
01 (*n* 30)	1⋅00	1⋅00	1⋅00	1⋅00
02 (*n* 54)	0⋅64 (0⋅29–1⋅46)	2⋅82 (1⋅10–7⋅26)	0⋅52 (0⋅21–1⋅28)	0⋅71 (0⋅33–1⋅49)
03 (*n* 25)	1⋅85 (0⋅62–5⋅50)	1⋅37 (0⋅48–3⋅92)	0⋅50 (0⋅17–1⋅47)	1⋅17 (0⋅47–2⋅92)
04 (*n* 81)	0⋅15 (0⋅07–0⋅32)	0⋅27 (0⋅12–0⋅63)	6⋅16 (2⋅45–15⋅49)	0⋅79 (0⋅38–1⋅64)
05 (*n* 66)	0⋅15 (0⋅06–0⋅35)	0⋅04 (0⋅02–0⋅11)	0⋅51 (0⋅21–1⋅26)	0⋅25 (0⋅12–0⋅55)

a Ordered logistic regression – odds of being in the third tertile of dietary patterns compared with the reference category.

b Family income per capita – divided in tertiles according to minimum wage effective in 2014 – of R$724⋅00/US$321⋅80^(25)^.

c Nutritional status regarding body mass index by age (BMI/A).

* *P* < 0⋅05.

** *P* < 0⋅01.

*** *P* < 0⋅001.

At homes, there was an association between ‘traditional’ and ‘milk and cereals’ patterns and the age of the child and current breast-feeding situation. Also, the ‘less healthy’ pattern showed an association with the age of the child, current breast-feeding situation and age of the mother, whereas the ‘mixed’ pattern was associated with the age of the child and education level of the mother. Concerning the age, children older than 12 months also presented higher scores for all dietary patterns identified at home. Besides, breastfed children showed lower scores for ‘traditional’, ‘less healthy’ and ‘milk and cereals’ patterns. Children, which mothers presented lower levels of education, had lower scores for the ‘mixed’ pattern. Finally, children with younger mothers presented higher scores for the ‘less healthy’ pattern (Table 5).

**Table 5.** Distribution of factor scores related to dietary patterns observed at home according to socio-economic, demographic variables and nutritional status for children under 2 years of age in municipal childcare centres in Guaratuba, Paraná, Brazil, 2014 (Odds ratio and confidence interval; statistical significance *P* < 0⋅05)

Variables	Pattern 1^a^ ‘Traditional’ Odds ratio (95 % confidence interval)	Pattern 2^a^ ‘Less healthful’ Odds ratio (95 % confidence interval)	Pattern 3^a^ ‘Milk and cereals’ Odds ratio (95 % confidence interval)	Pattern 4^a^ ‘Mixed'Odds ratio (95 % confidence interval)
Sex	*P* 0⋅826	*P* 0⋅826	*P* 0⋅245	*P* 0⋅212
Male (*n* 146)	1⋅00	1⋅00	1⋅00	1⋅00
Female (*n* 110)	1⋅05 (0⋅67–1⋅66)	1⋅05 (0⋅67–1⋅66)	0⋅76 (0⋅49–1⋅20)	0⋅75 (0⋅48–1⋅18)
Age of the child (months)	***P 0⋅000	***P 0⋅000	*P 0⋅045	***P 0⋅000
<12 (*n* 74)	1⋅00	1⋅00	1⋅00	1⋅00
≥12 (*n* 182)	7⋅56 (4⋅26–13⋅42)	7⋅34 (4⋅16–12⋅95)	1⋅68 (1⋅01–2⋅79)	2⋅77 (1⋅62–4⋅72)
Birth weight (g)	*P* 0⋅372	*P* 0⋅851	*P* 0⋅592	*P* 0⋅074
<2500 (*n* 20)	1⋅00	1⋅00	1⋅00	1⋅00
≥2500 and < 4000 (*n* 205)	1⋅35 (0⋅61–2⋅99)	0⋅85 (0⋅35–2,09)	1⋅20 (0⋅55–2⋅61)	1⋅15 (0⋅50–2⋅62)
≥4000 (*n* 25)	1⋅62 (0⋅56–4⋅63)	1⋅06 (0⋅34–3⋅28)	1⋅33 (0⋅46–3⋅86)	2⋅52 (0⋅86–7⋅38)
Current breast-feeding situation	**P 0⋅005	*P 0⋅010	***P 0⋅000	*P* 0⋅743
Yes (*n* 88)	1⋅00	1⋅00	1⋅00	1⋅00
No (*n* 162)	2⋅03 (1⋅25–3⋅32)	1⋅90 (1⋅17–3⋅09)	15⋅67 (8⋅50–28⋅89)	1⋅08 (0⋅67–1⋅76)
Age of the mother (years)	*P* 0⋅455	**P 0⋅004	*P* 0⋅409	*P* 0⋅908
≤20 (*n* 51)	1⋅00	1⋅00	1⋅00	1⋅00
>20 and ≤ 30 (*n* 118)	1⋅47 (0⋅80–2⋅69)	0⋅52 (0⋅28–0⋅96)	0⋅68 (0⋅36–1⋅25)	1⋅67 (0⋅92–3⋅01)
>30 (*n* 83)	0⋅87 (0⋅46–1⋅64)	0⋅37 (0⋅19–0⋅71)	0⋅72 (0⋅38–1⋅38)	1⋅11 (0⋅59–2⋅09)
Education level of the mother (years)	*P* 0⋅494	*P* 0⋅633	*P* 0⋅266	**P 0⋅001
<8 (*n* 57)	1⋅00	1⋅00	1⋅00	1⋅00
≥8 and < 11 (*n* 77)	0⋅90 (0⋅48–1⋅70)	1⋅05 (0⋅56–1⋅98)	1⋅24 (0⋅65–2⋅38)	2⋅43 (1⋅28–4⋅64)
≥11 (*n* 118)	1⋅17 (0⋅65–2⋅10)	0⋅89 (0⋅50–1⋅59)	1⋅40 (0⋅78–2⋅52)	2⋅83 (1⋅55–5⋅15)
Family income per capita^b^	*P* 0⋅144	*P* 0⋅501	*P* 0⋅469	*P* 0⋅353
≤0⋅35 (*n* 74)	1⋅00	1⋅00	1⋅00	1⋅00
>0⋅35 and < 0⋅70 (*n* 77)	0⋅78 (0⋅43–1⋅39)	0⋅77 (0⋅43–1⋅38)	1⋅39 (0⋅77–2⋅51)	1⋅24 (0⋅68–2⋅24)
≥0⋅70 (*n* 68)	0⋅63 (0⋅34–1⋅17)	0⋅82 (0⋅45–1⋅49)	1⋅26 (0⋅68–2⋅33)	0⋅75 (0⋅41–1⋅37)
Nutritional status^c^	*P* 0⋅773	*P* 0⋅706	*P* 0⋅559	*P* 0⋅507
Underweight (*n* 4)	1⋅00	1⋅00	1⋅00	1⋅00
Normal weight (*n* 148)	1⋅77 (0⋅26–11⋅91)	2⋅74 (0⋅46–16⋅38)	0⋅14 (0⋅02–1⋅35)	0⋅53 (0⋅08–3⋅53)
Overweight (*n* 53)	2⋅77 (0⋅40–19⋅44)	3⋅68 (0⋅59–23⋅04)	0⋅16 (0⋅02–1⋅56)	0⋅66 (0⋅09–4⋅62)
Obesity (*n* 46)	1⋅29 (0⋅18–9⋅14)	1⋅92 (0⋅30–12⋅12)	0⋅13 (0⋅01–1⋅30)	0⋅41 (0⋅06–2⋅87)
Childcare centres	*P 0⋅044	*P* 0⋅187	*P* 0⋅552	*P* 0⋅858
01 (*n* 30)	1⋅00	1⋅00	1⋅00	1⋅00
02 (*n* 54)	0⋅93 (0⋅40–2⋅15)	0⋅50 (0⋅22–1⋅15)	1⋅19 (0⋅50–2⋅81)	1⋅85 (0⋅80–4⋅27)
03 (*n* 25)	1⋅79 (0⋅65–4⋅92)	1⋅02 (0⋅38–2⋅69)	1⋅09 (0⋅40–2⋅97)	2⋅20 (0⋅81–5⋅99)
04 (*n* 81)	0⋅62 (0⋅28–1⋅39)	0⋅51 (0⋅23–1⋅09)	1⋅33 (0⋅59–2⋅98)	0⋅92 (0⋅43–1⋅97)
05 (*n* 66)	0⋅57 (0⋅25–1⋅30)	0⋅52 (0⋅23–1⋅15)	0⋅82 (0⋅36–1⋅87)	1⋅61 (0⋅72–3⋅59)

a Ordered logistic regression – odds of being in the third tertile of dietary patterns compared with the reference category.

b Family income per capita – divided in tertiles according to minimum wage effective in 2014 – of R$724⋅00/US$321⋅80^(25)^.

c Nutritional status regarding body mass index by age (BMI/A).

* *P* < 0⋅05.

** *P* < 0⋅01.

*** *P* < 0⋅001.

## Discussion

In the present sample of south Brazilian children under 2 years, four dietary patterns were identified in childcare centres and at homes. These dietary patterns were associated with socio-economic/demographic variables and nutritional status. The number of dietary patterns reached in this study was similar to the one found by Wen *et al.*^(11)^ in a study with American children of 6 and 12 months of age. Aside from these finding, four dietary patterns were found by Kristiansen *et al.*^(34)^, in a study that followed Norwegian children of 24 months of age, and by Lim *et al.*^(4)^, in which the consumption of Singapore's children aged from 6 to 12 months was evaluated.

The ‘traditional’ pattern, characterised by the presence of rice and beans, was common in both environments. Other Brazilian studies with children have also found the consumption of a similar traditional pattern^(22,35,36)^. Internationally, traditional patterns for children younger than 2 years of age have been identified and are basically composed of typical foods, thus differing from the Brazilian patterns in terms of food composition. Traditional patterns of European countries, such as Denmark, England and Norway, have been characterised by diverse foods, including potatoes, vegetables, meats, eggs, fish, breads, butter, sauces and puddings^(2,8,34,37,38)^. In addition, traditional patterns of Singapore, an Asian country, are based on pasta, eggs, seafood, dry fruits and tofu^(4)^. These results support the theory that dietary patterns are influenced by geographic localisation, local culture and ethnicity^(39)^.

The first pattern derived from the childcare centres was ‘snacks’, which gave rise to the largest variance value (27⋅5 %). This pattern is composed of foods that are commonly consumed between the main meals, do not require long preparation time, and are served in the school environment as breakfast or an afternoon snack, such as biscuits and breads, margarine and butter, coffee and teas, and fruits. Other studies carried out with children have also identified similar patterns called ‘snacks’^(35,36,40)^. However, in some studies, ‘snacks’ is composed of less healthy foods, for example, candies and soft drinks, and for this reason, this pattern has a negative connotation^(22,41)^.

On the other hand, the ‘less healthy’ pattern – composed of foods rich in sugar, sodium and fat, such as dainties, sugar, sugar drinks, processed meats, pasta and salty, and margarine and similar – was not identified in the childcare centres. This observation could be explained by the fact that there is a law that establishes prohibitions and limitations in relation to the nutritional quality of foods served in childcare centres. The Brazilian legislation related to scholar feeding allows the inclusion of only two portions of candies or sweets per week, limiting the daily amount of added sugar, fat and sodium and prohibiting the acquisition of artificial refreshments and soft drinks. This legislation explains the reason why ‘sugar drinks’ group was not found in any of the patterns identified in this environment^(42)^.

Conversely, the ‘less healthy’ pattern was found at homes. The Brazilian food guideline for children under 2 years of age recommends avoiding such types of foods during the first years of life, since they may lead to health issues, such as anaemia, overweight and allergies. Such foods may also hinder the acceptance of healthy ones, for example, vegetables, which are essential nutrient sources for proper growth and child development^(43)^. Similar less healthy dietary patterns have also been identified in other studies with children under 2 years of age ^(6–8,10–12,34,37,38,44–47)^.

Furthermore, another pattern identified in childcare centres was the ‘nutritive’ one that is composed of foods rich in vitamins, minerals and fibres, such as natural juice and vegetables and it had negative loading for breakfast cereals. The ‘nutritive’ pattern is similar to the ‘mixed’ one identified at homes, and the similarity is due to the presence of tubercles, roots and cereals, and vegetables. However, the ‘mixed’ pattern is also composed of coffee, teas and formula, and it was the only one that presented a weak internal consistency (Cronbach's α <0⋅6).

It was still possible to observe a ‘milk and cereals’ pattern at homes. Breakfast cereals, mainly composed of industrialised products with added sugar, are usually offered in milk-based preparations and may often substitute main meals composed of different alimentary groups, such as vegetables, meats and tubercles. Moreover, the ‘milk’ group was predominantly constituted by cow's milk, which is not suitable for children less than 1-year-old due to its high dose of proteins and electrolytes, causing impairment in the absorption of micronutrients, thus leading to allergies and anaemia due to iron deficiency^(43)^. Although most of the children in this study are over 12 months of age and the recommendation for this age is to offer the same feeding from the family, the food consumption of this pattern was frequent for most of the children. On the other hand, this pattern presented negative loading for breast milk, indicating that the children who were breastfed had less consumption of milk and breakfast cereals.

To the best of our knowledge, only one study described dietary patterns of children inside and outside school. It corresponds to a Brazilian multicentre study, which also identified four dietary patterns in the school environment, named ‘traditional’, ‘dual’, ‘snacks’ and ‘bread with butter’ patterns. The main difference compared with this study is that it evaluated both public and private schools, in which children were more way to consume a ‘snacks’ pattern consisting of foods with lower nutritional quality^(22)^.

Children over 12 months of age presented higher scores for all dietary patterns identified in both environments. A possible explanation is that children over 12 months of age consume foods independently from their parents and caregivers, thus gain access to a greater variety of foods. Besides, only 26⋅4 % of such children were breastfed, whereas 53⋅3 % of the children under 12 months of age were still being breastfed, which can result in a lower consumption of others types of foods in the breastfed group. This is supported by the at-home observations of this study, in which breastfed children presented lower scores for the ‘traditional’, ‘less healthy’ and ‘milk and cereals’ patterns. Studies that investigated the association between dietary patterns and breast-feeding duration found higher scores for the ‘healthy’ pattern not only for longer breast-feeding periods^(45)^ but also for children exclusively breastfed in the first 4 months^(46)^. Furthermore, it was observed higher scores for the ‘unhealthy’ pattern for precocious weans^(45,47)^, and lower scores were associated with longer breast-feeding periods^(34)^.

There was an association between the age of the mother and dietary patterns only at homes. The younger the mother, the higher the scores for the ‘less healthy’ pattern. The outcomes found at homes agree with other studies^(38,44,45,48)^. Younger mothers can be less inclined to cook and have less knowledge on healthy eating habits^(48)^ enabling them to offer easy to prepare foods^(4)^, which might contribute to a higher consumption of industrialised foods.

It was possible to verify an association between the educational level of the mother and dietary patterns only at homes, in which children whose mothers had lower levels of education presented lower scores for the ‘mixed’ pattern. Different studies carried out with children under 2 years of age have found an association between higher adherence to healthier dietary patterns and a higher level of education of the mother^(4,11,34,38,44,45,48,49)^. Mothers with a lower level of education may not have the necessary knowledge on appropriate eating habits, which might lead to less healthy eating habits in children^(11)^.

In the childcare centres, it was possible to verify an association between lower family incomes per capita and higher scores related to the ‘snacks’ and ‘traditional’ patterns. In other studies, in which associations between the dietary patterns of children under 2 years of age and the familiar income were investigated, it was observed an association between lower family incomes and an increased adherence to the ‘less healthy’ pattern^(11,46,47)^, as well as an association between higher family incomes and an increased adherence of the ‘healthy’ pattern^(4,11,48)^.

Obese children showed lower scores for all dietary patterns identified in childcare centres. A possible explanation for such a result may be the lack of consumption in the childcare centres compensated by the high energy density foods consumed at homes. In a previous research conducted with the same population of children investigated in this study, it was noticed that food consumption at homes corresponded to the higher amount of energy and nutrient intakes^(23)^. It is common for children to adjust their energy intake throughout the meals, keeping it constant^(50)^. Therefore, the acceptance of foods offered in the childcare centres may decrease due to greater consumption at homes^(51)^. Other studies have also observed the association between higher Z-scores of BMI and lower adherence to different dietary patterns identified as ‘Transition food’^(2)^, ‘Family food’ and ‘Health-conscious’^(52)^.

In addition, adherence to dietary patterns was different depending on the childcare centres investigated, probably due to differences in their menus. Possible reasons can be the differences in the period during which data were collected in each place, or since the food suppliers do not follow the menu proposed by the nutritionists responsible for the scholar eating programme. According to Issa *et al.*^(53)^, not following the menu instructions is a common practice observed in the school environment. The practice of foods on the menu not being served is explained mainly because of a lack of stock control or failure in the supply.

This study, however, has some limitations. The transversal drawing is insufficient to infer causal relations between dietary patterns and the associated variables. The analysis of dietary patterns involves some subjective decisions (biases), which may influence the result of the dietary pattern, such as the formation of food groups, selected methods to generate dietary patterns, the number of factors to be extracted and the denomination of the identified patterns. Some patterns remained related to each other, even though they have been used in the orthogonal rotation recommended for favouring the identification of less correlated patterns. The application of the oblique rotation method also presented a similar result. Another aspect was the impossibility to carry out independent analyses according to age due to the small number of children under 1 year of age, and therefore, values were below the estimated minimum number of children per food groups. Thus, it was not possible to introduce multivariate analysis for age^(54)^. This age range would be important to analyse eating habits according to age and would show variations in the type and amount of foods consumed by the children, especially during their transition phase. To reduce the limitations of the subjective decisions, scientific literature was reviewed in order to choose the most used methods in other studies, which performed factorial analysis and Varimax rotation. The number of retained factors was based on the combination of quantitative criteria and interpretability of the components in agreement with Fransen *et al.*^(55)^.

The strengths of this study include the method of weighed food recordings in order to obtain data related to food consumption in the childcare centres, as it is considered one of the most accurate methods^(16)^. Also, the inclusion of all children under 2 years of age were enrolled and attended all childcare centres during the period of collection, reinforcing the internal validity of the data.

## Conclusions

We identified four dietary patterns in each environment. In both environments, the ‘traditional’ pattern was observed; only at homes, it was possible to find a ‘less healthy’ pattern. Adherence to dietary patterns was associated with socio-economic, demographic variables and nutritional status. Obese children showed lower scores for all patterns in the childcare centres. There was an association between maternal age and higher adherence to the ‘less healthy’ pattern, as well as an association between the level of education of the mother and lower adherence to the ‘mixed’ pattern, and between lower familiar income per capita and higher adherence to the ‘snacks’ and ‘traditional’ patterns in childcare centres. It is noteworthy that in the present study, it was not identified neither the ‘less healthy’ pattern nor the sugar drink food group in childcare centres. This fact may be explained by the impact of the national public scholar feeding policy, which establishes rules regarding the purchase and supply of some food groups. Given such results, it is relevant to create an intersectional dialogue between health and education in order to offer educative actions of health promotion in the school and at home. In addition, further studies are needed, especially those with longitudinal designs, to enable the monitoring of dietary patterns.
